# Data-scientific validation of prediction models for the controlled syntheses of exfoliated nanosheets†

**DOI:** 10.1039/d5na00215j

**Published:** 2025-06-04

**Authors:** Yuka Kitamura, Yuki Namiuchi, Hiroaki Imai, Yasuhiko Igarashi, Yuya Oaki

**Affiliations:** a Department of Applied Chemistry, Faculty of Science and Technology, Keio University 3-14-1 Hiyoshi, Kohoku-ku Yokohama 223-8522 Japan oakiyuya@applc.keio.ac.jp; b Institute of Engineering, Information and Systems, University of Tsukuba 1-1-1 Tennodai Tsukuba 305-8573 Japan igayasu1219@cs.tsukuba.ac.jp

## Abstract

Exfoliated nanosheets have attracted considerable interest as two-dimensional (2D) building blocks. In general, the yield, size, and size distribution of the exfoliated nanosheets cannot be easily controlled or predicted because of the complexity in the processes. Our group studied the prediction models of the yield, size, and size distribution based on the small experimental data available. Sparse modeling for small data (SpM-S) combining machine learning (ML) and chemical insight was used for the construction of predictors. In SpM-S, the weight diagram visualizing the significance of explanatory variables plays an important role in variable selection to construct the models. However, the processes of variable selection were not validated in a data-scientific manner. In the present work, the significance of data size, visualization method, and chemical insight for variable selection was studied to validate the processes of model construction. The data size had a lower limit to extract appropriate descriptors. The weight diagram had an appropriate visualizing range for variable selection. Chemical insight as domain knowledge supplemented the limitation caused by the data size. These studies indicated that SpM-S can be applied to construct predictors, straightforward linear regression models, for the controlled syntheses of other 2D materials, even based on small data.

## Abstract 2

Data-scientific validation is demonstrated on prediction models for the yield and size control of 2D materials. The significance of data size, descriptor visualizing method, and domain knowledge is studied in sparse modeling for small data.

## Graphical abstract

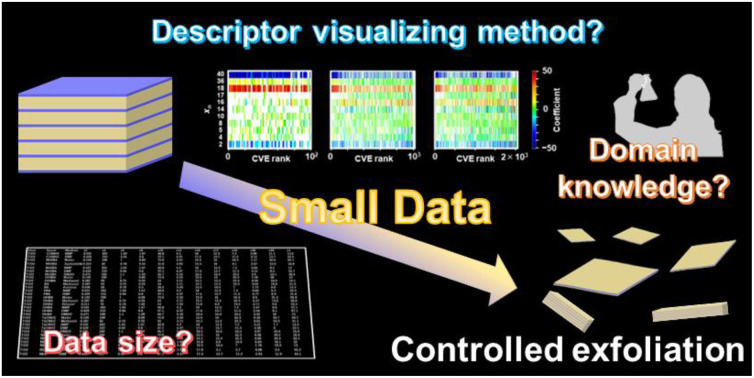

## 1. Introduction

**Fig. 1:**
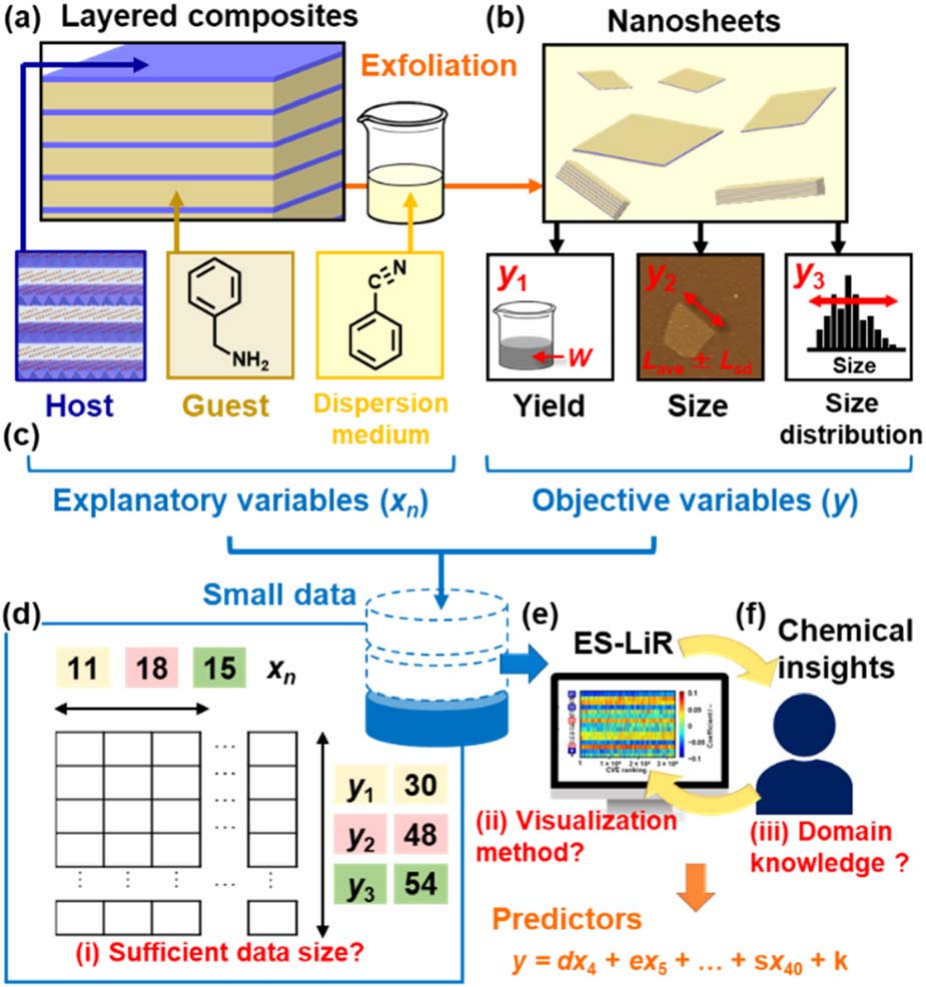
Overview of the small-data-driven exfoliation experiments. (a) Precursor layered composites of inorganic hosts and organic guests and their exfoliation in organic dispersion media. (b) Yield (*y*_1_), lateral size (*y*_2_), and size distribution (*y*_3_) of the surface-functionalized nanosheets. (c) Explanatory variables (*x*_*n*_) and objective variables (*y*: *y*_1_, *y*_2_, *y*_3_). (d) Small datasets and their contents. (e and f) Variable selection using ES-LiR (e) and our chemical insight (f) as ML and domain knowledge, respectively, for the construction of predictors.

Liquid-phase exfoliation is a general method used to obtain 2D materials, including monolayers and few-layers.^1–9^ Various precursor layered materials can be exfoliated into nanosheets. However, the exfoliation behavior cannot be easily controlled because of the complex and random downsizing processes in both the lateral and thickness directions. For example, the yield, size, and size distribution of the exfoliated nanosheets are not easily controlled by specific parameters based only on professional experience. In recent years, data-scientific approaches have been applied to the field of 2D materials for their design, synthesis, and characterization.^10–14^ Our group has focused on the construction of prediction models to control the yield, size, and size distribution of surface-modified nanosheets exfoliated from precursor layered composites (Fig. 1a–c).^4,10,15–18^ The precursor layered composites are typically synthesized by the intercalation of the guest organic molecules in the interlayer space of host layered transition-metal oxides (Fig. 1a). The surface-modified nanosheets are then obtained through exfoliation by the dispersion of the layered composites in organic dispersion media (Fig. 1b). Although the exfoliation behavior could be changed by varying the combinations of the host, guest, and medium, their effects on the yield, size, and size distribution of the nanosheets were unclear. In recent years, prediction models for the control of these parameters have been constructed by combining machine learning and our chemical insight on small experimental data.^4,10,15–18^ Moreover, controlled syntheses have been achieved using the predictors in a limited number of experiments. However, the model construction processes have not yet been fully studied in a data-scientific manner. If the model construction processes could be validated, then similar predictors could be constructed by SpM-S for various other 2D materials.

Data-driven approaches have been used in a broad range of chemistry and materials science areas.^19–27^ For instance, the combination of big data, machine learning (ML), and a robotic system has been studied to develop fully automated AI chemists.^28–32^ These AI-oriented methods are supported by the availability of a sufficient size of data for the ML. However, a sufficient size of data is not always available for all experimental systems. For example, big data is not efficiently collected from conventional experimental works including batch processes. Specific methods are thus required to apply ML to small data.

ML for small data has been increasingly studied in recent years.^33–45^ Specific approaches, such as transfer learning, have been developed for the use of small data.^33–45^ However, the interpretability and generalizability are lower for modeling based on complex modeling algorithms. Recent reports have indicated the significance of domain knowledge and the use of simple regression models.^43–45^ Our group has focused on sparse modeling (SpM), a method for describing whole high-dimensional data by a small number of significant descriptors.^46–48^ SpM has already been applied in a variety of fields, such as image compression and materials science.^15–18,49–54^ We have studied SpM for small data (SpM-S) combining ML and domain knowledge.^10,45^ The method was applied to controlled the synthesis of nanosheets and the exploration of electrode active materials based on small data.^15–18,51–54^ In SpM-S, the descriptors are extracted from a small training dataset using a ML algorithm, and an exhaustive search with linear regression (ES-LiR), as mentioned later (Fig. 1c–e). Then, the descriptors are further selected based on our domain knowledge as chemists (Fig. 1f). A straightforward linear regression model is then constructed using the selected descriptors. In our previous work,^45^ the prediction results of SpM-S combining linear regression and our chemical insight were compared with those obtained from other linear and nonlinear algorithms, such as least absolute shrinkage and selection operate (LASSO) and neural network regression (NN-R), in terms of the accuracy, interpretability, and generalizability, especially for small data. Although nonlinear algorithms, such as NN-R, generally exhibit high expressive power, they tend to overfit small chemical experimental datasets because of the insufficient generalizability.^45^ However, the processes remain unclear with problems persisting regarding the variable selection, one of the significant steps for modeling (problems (i)–(iii) in Fig. 1d–f) regarding the required data size (problem (i)), visualization method of the weight diagram (problem (ii)), and the significance of domain knowledge (problem (iii)). The present study aimed to solve these problems to improve the understanding of SpM-S. The results indicated that similar predictors could be constructed by SpM-S based on small experimental data for various other 2D materials.

## 2. Results and discussion

### 2.1. Prediction models for exfoliated nanosheets

The prediction models of the yield, size, and size distribution were constructed using the small training datasets I–III in our previous works, respectively (Fig. 1 and Tables S1–S3 in the ESI†).^16–18,45^ The objective variables (*y*) were the yield (*y*_1_), lateral size (*y*_2_), and lateral-size distribution (*y*_3_) of the nanosheets (Fig. 1a and b). The yield (*y*_1_ = 100 × *W*/*W*_0_) was calculated from the weight of the collected nanosheets with the filtration (*W*) and that of the precursor layered materials (*W*_0_).^16^ The average lateral size (*L*_ave_) and its standard deviation (*L*_sd_) were measured by dynamic light scattering as for a high-throughput method. The lateral size (*y*_2_ = *R*_L_) is defined as its reduction rate *R*_*L*_ = *L*_ave_/*L*_0_,^17^ where *L*_0_ is the lateral size of the precursor layered materials. The size distribution (*y*_3_ = *L*_CV_), polydispersity, is represented by the coefficient of variation about the lateral size (*L*_CV_ = *L*_sd_/*L*_ave_).^18^

The explanatory variables (*x*_*n*_: *n* = 1–41), such as the physicochemical parameters of the guests and media, were the related physicochemical parameters selected by our chemical insights (Table 1). In the total 41*x*_*n*_, the selected *x*_*n*_ were used as the potential descriptors for *y*_1_–*y*_3_. The datasets contained the following numbers of *y* and *x*_*n*_ (Table 1): 30*y*_1_ and 11*x*_*n*_ (*n* = 2, 4, 5, 8, 10, 14, 16–18, 36, 40) (dataset I), 48*y*_2_ and 18*x*_*n*_ (*n* = 1, 3–5, 14–21, 30–32, 34, 36, 40) (dataset II), 54*y*_3_ and 15*x*_*n*_ (*n* = 4, 8, 10, 13, 14, 16–18, 21, 30–32, 36, 40, 41) (dataset III) (Fig. 1c and d). In our previous works, the descriptors were extracted from *x*_*n*_ by SpM using ES-LiR (Fig. 1d and e). Then, the descriptors were further selected with the assistance of our chemical insights (Fig. 1e and f). The linear regression models eqn (1)–(3) were constructed using the selected two to eight *x*_*n*_.^16–18^

1 *y*_1_ = 35.00*x*_3_ − 32.33*x*_5_ + 34.07

2 *y*_2_ = −0.159*x*_3_ − 0.096*x*_4_ + 0.257*x*_7_ − 0.017*x*_8_ − 0.018*x*_10_ + 0.028*x*_13_ − 0.050*x*_14_ + 0.061*x*_18_ + 0.267

3 *y*_3_ = −0.0599*x*_7_ + 0.0802*x*_9_ + 0.0699*x*_20_ − 0.0681*x*_28_ − 0.0623*x*_37_ + 0.266

As the coefficients are converted to the normalized frequency distribution with mean 0 and standard deviation 1 for the variables in each model, the weight of the contribution is represented by the coefficients. In the present work, the processes of the variable selection were studied to validate the models themselves and their construction processes.

**Table 1:** List of *x*_*n*_ (*n* = 1–41) for *y*_1_, *y*_2_, and *y*_3_ (ref. 45)

*n*	Parameters	*x*_*n*_ for
**Dispersion media**		
1	Molecular weight	*y*_1_, *y*_2_, *y*_3_
2	Molecular length^b^	*y*_1_
3	Melting point^a^	*y*_1_, *y*_2_, *y*_3_
4	Boiling point^a^	*y*_1_, *y*_2_, *y*_3_
5	Density^a^	*y*_1_, *y*_2_, *y*_3_
6	Relative permittivity^a^	*y*_1_, *y*_2_, *y*_3_
7	Vapor pressure^a^	*y*_1_, *y*_2_, *y*_3_
8	Viscosity^a^	*y*_1_, *y*_2_, *y*_3_
9	Refractive index^a^	*y*_1_, *y*_2_, *y*_3_
10	Surface tension^a^	*y*_1_, *y*_2_, *y*_3_
11	Heat capacity^b^	*y*_1_, *y*_2_, *y*_3_
12	Entropy^b^	*y*_1_, *y*_2_, *y*_3_
13	Enthalpy^b^	*y*_1_, *y*_2_, *y*_3_
14	Dipole moment^b^	*y*_1_, *y*_2_, *y*_3_
15	Polarizability^b^	*y*_1_, *y*_2_, *y*_3_
16	HSP-dispersion^b^	*y*_1_, *y*_2_, *y*_3_
17	HSP-polarity^b^	*y*_1_, *y*_2_, *y*_3_
18	HSP-hydrogen bonding^b^	*y*_1_, *y*_2_, *y*_3_

**Guest molecules**		
19	Molecular weight	*y*_1_, *y*_2_, *y*_3_
20	Polarizability^b^	*y*_1_, *y*_2_, *y*_3_
21	Dipole moment^b^	*y*_1_, *y*_2_, *y*_3_
22	Heat capacity^b^	*y*_1_, *y*_2_, *y*_3_
23	Entropy^b^	*y*_1_, *y*_2_, *y*_3_
24	Enthalpy^b^	*y*_1_, *y*_2_, *y*_3_
25	Molecular length^b^	*y*_1_
26	Layer distance^c^	*y*_1_, *y*_2_, *y*_3_
27	Layer distance expansion^c^	*y*_3_
28	Composition (*x*)^c^	*y*_1_, *y*_2_
29	Interlayer density^c^	*y*_1_, *y*_2_
30	HSP-dispersion terms^b^	*y*_1_, *y*_2_, *y*_3_
31	HSP-polarity terms^b^	*y*_1_, *y*_2_, *y*_3_
32	HSP-hydrogen bonding terms^b^	*y*_1_, *y*_2_, *y*_3_

**Guest-medium combinations**		
33	Δ polarizability (=*x*_15_ − *x*_20_)^b^	*y*_3_
34	Δ polarizability (=|*x*_33_|)^b^	*y*_1_, *y*_2_, *y*_3_
35	Δ dipole moment (=*x*_14_ − *x*_21_)^b^	*y*_3_
36	Δ dipole moment (=|*x*_35_|)^b^	*y*_1_, *y*_2_, *y*_3_
37	Product of dipole moment (=*x*_14_ × *x*_21_)^b^	*y*_3_
38	Δ heat capacity (=*x*_11_ − *x*_22_)^b^	*y*_3_
39	Δ heat capacity (=|*x*_38_|)^b^	*y*_1_, *y*_2_, *y*_3_
40	HSP distance^b^	*y*_1_, *y*_2_, *y*_3_

**Host**		
41	Bulk size^c^	*y*_3_

a Literature data.

b Calculation data.

c Experimental data.

### 2.2. Effects of the data size on the extraction of the descriptors

**Fig. 2:**
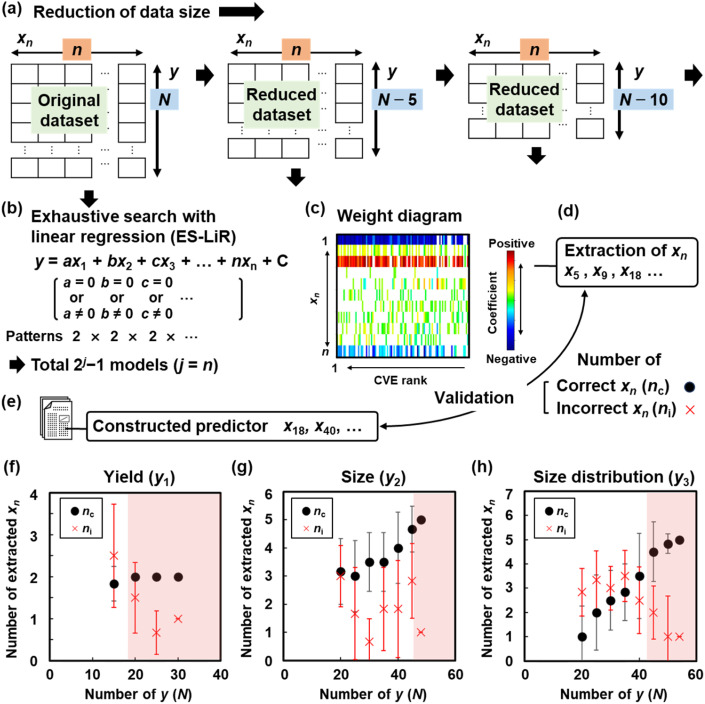
Effect of data size on variable selection. (a) Reduction of the data size, the number of *y* (*N*). (b) Scheme of ES-LiR. (c) Weight diagram visually representing the contribution of each *x*_*n*_. (d) Extraction of *x*_*n*_ from the weight diagrams with reducing *N* as shown in panels (a–c). (e) Counting *n*_c_ and *n*_i_ based on comparison of the extracted *x*_*n*_ with that in the already constructed predictors in our previous works as the correct models. (f–h) Summary of *n*_c_ and *n*_i_ with reducing *N* for *y*_1_ (f), *y*_2_ (g), and *y*_3_ (h).

The extractability of the descriptors generally depends on the data size. In the present work, the descriptors were extracted with reducing the data size in datasets I–III to study whether the data size was sufficient for the extraction of the descriptors (Fig. 2). The detailed procedure is described in the ESI.† The number of *y* (*N*) was decreased step-by-step (Fig. 2a and Tables S1–S3 in the ESI†). For example, the original 30*y*_1_ was reduced to 25*y*_1_ with the random subtraction of five *y*_1_. Then, reduced datasets with 20*y*_1_ were prepared with a further subtraction of 5*y*_1_. The five subtracting data items were randomly selected and six different datasets were prepared at each *N*. In this manner, reduced datasets were prepared for each of *y*_1_, *y*_2_, and *y*_3_.

The extractability of the descriptors was then studied using the reduced datasets. Weight diagrams were prepared by ES-LiR. Linear regression models were prepared by all the possible combinations of *x*_*n*_ (*n* = 1, 2, …, *j*), *i.e*., total 2^*j*^ − 1 combinations, on each dataset with five-fold cross-validation (Fig. 2b). As pointed out, Hastie *et al.* suggested *“*ten-fold cross-validation achieves an acceptable trade-off between bias and variance,”^55^ and this has since become standard practice.^56^ However, both 5- and 10-fold cross-validation are generally recognized as appropriate choices due to their superior stability compared to leave-one-out cross-validation (LOOCV). Despite its theoretical appeal, LOOCV exhibits high variance in performance estimates and is thus less reliable for model selection.^57^ In our study, five-fold cross-validation was used in terms of its computational efficiency and as standard practice. After the models were sorted in ascending order of cross-validation error (CVE), *i.e.,* CVE ranking, the values of the coefficients for each regression model were visualized in the weight diagram (Fig. 2c). Conventional ML algorithms require tuning the hyperparameters to optimize the models.^58,59^ Whereas, as ES-LiR just prepares all the possible linear regression models, the modeling method has no hyperparameters to be tuned compared with other ML algorithms. The contribution of each *x*_*n*_ was color-coded by the magnitude of the coefficients with their positive and negative values. The more deeply colored *x*_*n*_ with warmer and cooler colors have potential as more significantly contributed descriptors with the positive and negative correlations, respectively. The more densely colored *x*_*n*_ correspond to the more frequently used descriptors, implying a significant contribution to *y*. Weight diagrams were prepared for all the reduced datasets (Fig. S1–S3 in the ESI†). Based on the weight diagrams, we extracted *x*_*n*_ as the descriptors with reference to the deepness and density of the color (Fig. 2c and d). Here *x*_*n*_ in the already constructed models eqn (1)–(3) were assumed to be the true ones (Fig. 2e). If the visually extracted *x*_*n*_ from the weight diagram was found in the already constructed models, the extracted *x*_*n*_ here could be regarded as the correct ones. In contrast, the extracted *x*_*n*_ that were not found in the constructed models were regarded as incorrect ones (Fig. 2d and e). After the weight diagrams were prepared for the six different reduced datasets at each data size (*N*) (Fig. S1 in the ESI†), the numbers of correctly and incorrectly extracted *x*_*n*_ (*n*_c_ and *n*_i_, respectively) were counted and are summarized in Fig. 2f–h. The mean and standard deviation of *n*_c_ and *n*_i_ were calculated for the six different datasets.

When *N* was reduced, *n*_c_ decreased and *n*_i_ increased (Fig. 2f–h). Here the threshold *N* to extract the correct descriptors (*N*_min_) is defined as follows: the average *n*_i_ is less than two and *n*_c_ is more than 80% of the true *n*_c_ before the data reduction. The threshold data size to extract the correct *x*_*n*_, *N*_min_, was 20 for *y*_1_ (the original data size: *N*_0_ = 30), 45 for *y*_2_ (*N*_0_ = 48), and 45 for *y*_3_ (*N*_0_ = 54) (red-colored areas in Fig. 2f–h). These results indicate that the correct *x*_*n*_ can be extracted from the weight diagrams based on datasets with *N*_min_ < *N*. *N*_min_ can be regarded as the minimum required data size to extract the correct descriptors from the weight diagram. As *N*_min_ < *N*_0_ was achieved for *y*_1_, *y*_2_, and *y*_3_, the original datasets already had a sufficient data size for the model construction. Therefore, the generalizable *x*_*n*_ could be extracted from the weight diagrams even based on the small dataset at *N*_min_ < *N*. These results support the prediction models for the yield, size, and lateral size, and so eqn (1)–(3) were constructed with a sufficient size of data. Moreover, this data-reduction method can be applied to validate the sufficiency of the data size for the variable selection in small data.

### 2.3. Visualization method of weight diagrams

**Fig. 3:**
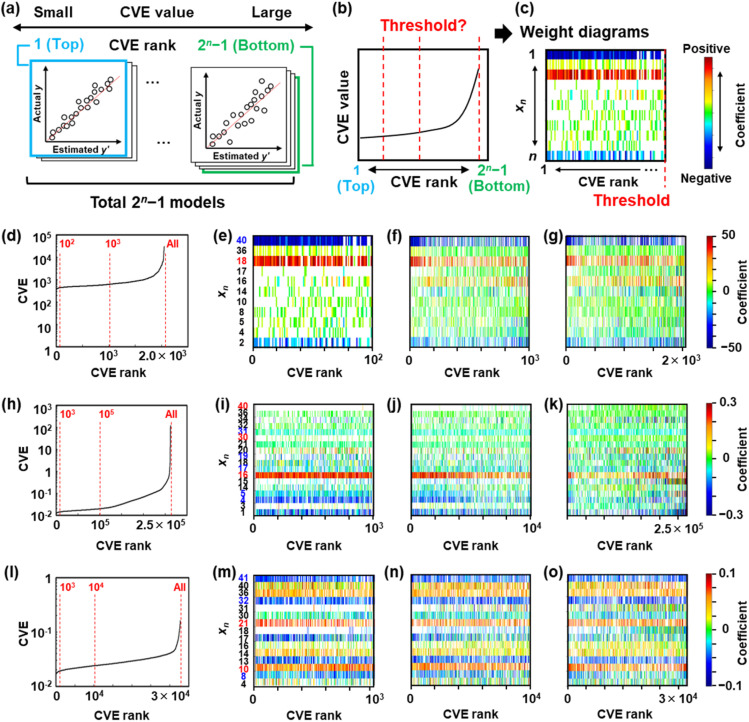
Visualization method of weight diagrams. (a) All possible linear regression models, 2^*n*^ − 1 patterns, sorted in an ascending order of the CVE rank. (b) Relationship between the CVE rank and value. (c) Preparation of the weight diagrams with the different threshold of the CVE rank. (d, h and l) Relationship between the CVE rank and values for *y*_1_ (d), *y*_2_ (h), *y*_3_ (l). (e–g) Weight diagrams of *y*_1_ within the top CVE rank 1.0 × 10^2^ (e), 1.0 × 10^3^ (f), and 2.0 × 10^3^ (g). (i–k) Weight diagrams of *y*_2_ within the top CVE rank 1.0 × 10^3^ (i), 1.0 × 10^4^ (j), and 2.6 × 10^5^ (k). (m–o) Weight diagrams of *y*_3_ within the top CVE rank 1.0 × 10^3^ (m), 1.0 × 10^4^ (n), and 3.3 × 10^4^ (o).

As discussed in Section 2.1, in SpM-S, the coefficients of *x*_*n*_ are visualized in the weight diagram based on the CVE values (Fig. 3a–c). In contrast, the other ML algorithms for SpM, such as LASSO and minimax concave penalty and penalized linear unbiased selection (MCP), only focus on a certain model with the smallest CVE values. Such algorithms raise concerns about the false extraction of descriptors, particularly in small data. ES-LiR visualizes a large number of models using the weight diagram in the ascending order of the CVE values. However, the preparation method of the weight diagram is somewhat arbitrary; in particular, the visualizing range of the CVE rank in the horizontal axis is arbitrary (Fig. 3b and c). If the appearance of the weight diagram is changed depending on the visualizing range, then different *x*_*n*_ can be extracted. Here the range displaying CVE rank in the horizontal axis was changed to study the effects on the appearance of the weight diagram for extractability of the descriptors (Fig. 3b and c).

The relationship between the CVE rank and CVE value was determined to visualize the increasing trend of the CVE value (Fig. 3b). Then, the weight diagrams were prepared within the different CVE ranks as the thresholds (Fig. 3c). The CVE values gradually increased with lowering the rank and then jumped near the bottom (Fig. 3d, h and l). As the datasets contained 11, 18, and 15*x*_*n*_ for *y*_1_, *y*_2_, and *y*_3_, respectively, the total number of exhaustively constructed models (2^*j*^ − 1 combinations) was 2.0 × 10^3^ for *y*_1_, 2.6 × 10^5^ for *y*_2_, and 3.3 × 10^4^ for *y*_3_. Whereas the correct *x*_*n*_ (*n* = 18, 40) were clearly visible in the weight diagram within the CVE rank 1.0 × 10^2^ for *y*_1_ (Fig. 3e), the weight diagrams became unclear in the ranks 1.0 × 10^3^ and 2.0 × 10^3^ (Fig. 3f and g). The correct *x*_*n*_ (*n* = 18, 40) could not be extracted from these unclear weight diagrams. Clear weight diagrams were observed in the ranks 1.0 × 10^4^ for *y*_2_ and 1.0 × 10^3^ for *y*_3_ (Fig. 3i, j and m). The visibility was lowered in the weight diagrams in the CVE ranks lower than 1.0 × 10^5^ for *y*_2_ and 1.0 × 10^4^ for *y*_3_ (Fig. 3k, n and o).

Based on these results, it could be seen that the visibility of the weight diagrams and extractability of the descriptors were changed by the range of the CVE rank. The weight diagrams within the CVE ranks about the top 10%, namely 10^2^ for *y*_1_, 10^4^ for *y*_2_, and 10^3^ for *y*_3_, allowed a clear extraction of the descriptors. The CVE ranks achieving one standard error rule were calculated to be 2.3 × 10^2^ for *y*_1_, 4.6 × 10^4^ for *y*_2_ and 2.0 × 10^3^ for *y*_3_. In the present work, the top 10% of the CVE rank was coincident with one standard error rule. Here, one standard error rule was used to estimate the range of the CVE ranks for visualization. The scheme means that all models having a CVE within one standard deviation of the minimum CVE were considered, resulting in the selection of approximately the top 10% of the CVE rankings. However, this coincide was not necessary. In general, the one standard error rule is used to optimize the regularization parameter (*λ*) in ML.^60^ When a larger penalty term is set, the one standard error rule is used to optimize *λ* instead of the minimum CVE value. These facts imply that a similar scheme can be applied to estimate the threshold for distinctly increasing the CVE by one standard error rule. In contrast, the visibility becomes unclear when the range was expanded to the CVE rank over the top 50%. Whereas the correct descriptors could be extracted from the clear weight diagram, the unclear weight diagram caused an extraction of the wrong descriptors and an oversight of the correct ones. As clear weight diagrams with the top 10% rank were used in our previous works,^16–18^ appropriate descriptors were extracted for the construction of the models in eqn (1)–(3).

### 2.4. Effects of the domain knowledge on the variable selection

As demonstrated in Sections 2.1 and 2.2, the appearance of the weight diagrams, such as the color density and intensity, easily varied by the data size and noise resulting from the small data. Therefore, our chemical insight as experimental scientists was used for the further selection and rejection of the descriptors in addition to the weight diagrams. Such domain knowledge facilitates robust modeling based on small data. As a reference, here an exhaustive search with Bayesian model averaging (ES-BMA) was used to extract the descriptors without the assistance of our domain knowledge. The variable selection using ES-BMA was then compared with that using ES-LiR combined with our domain knowledge.

**Fig. 4:**
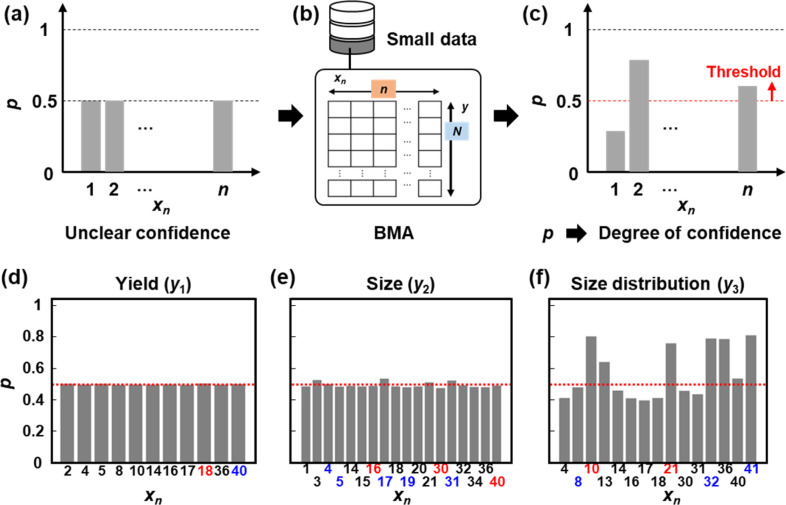
Variable selection using ES-BMA. (a) Probability (*p*) before ES-BMA. (b) BMA based on small data. (c) *p* after ES-BMA. (d) *p* of each *x*_*n*_ for *y*_1_ (d), *y*_2_ (e), *y*_3_ (f) after ES-BMA.

In ES-BMA, the probability of a descriptor (*p*) being the significant descriptor was 0.5 for all *x*_*n*_ at the initial state (Fig. 4a). The descriptors were not extracted because *p* = 0.5. The probability *p* of each *x*_*n*_ was calculated using the prediction accuracy and coefficient of the 2^*n*^ − 1 models prepared by ES-LiR (Fig. 4b and c). In ES-BMA, we consider the uncertainty for all 2^*n*^ combinations of variables and introduce a method for quantitatively evaluating the confidence level of variable selection using a weighted average of the model posterior probabilities, which is called Bayesian model averaging (BMA) (Fig. 4b).^61^ This method enables evaluating the confidence level of the variable selection and quantifying the importance evaluation of the features, whereas the processes quantitatively depend on the visibility of the weight diagram for ES-LiR. Furthermore, this approach quantitatively assesses the plausibility of the descriptors under the assumption of uniform prior knowledge without relying on the expertise of chemists. The summation over all combinations of indicator vectors can be calculated using the result of the exhaustive search, which is called ES-BMA.^61^

Fig. 4d–f shows the *p* of each *x*_*n*_. The descriptors for *y*_1_ and *y*_2_ were not extracted from the probability of each *x*_*n*_ because *p* was almost 0.5 (Fig. 4d and e). On the other hand, *x*_10_, *x*_13_, *x*_21_, *x*_32_, *x*_36_, *x*_40_, and *x*_41_ for *y*_3_ showed *p* > 0.5 (Fig. 4f). Four descriptors *x*_10_, *x*_21_, *x*_32_, and *x*_41_ of five correct *x*_*n*_ in eqn (3) were extractable by the *p* value based on ES-BMA. These results imply that the data size was insufficient to extract the true descriptors only using ES-BMA without the domain knowledge. The combination of ES-LiR and domain knowledge facilitated the extraction of the descriptors. In SpM-S, the domain knowledge contributed to being able to extract the descriptors and construct the models.

In SpM-S, professional experience and chemical insights, as domain knowledge, are mainly used in the processes of variable selection based on the weight diagram. Although the weight diagram indicated the strong contribution of certain variables, some variables were not used as the descriptors for modeling based on our chemical insight. For example, this scheme provides a more accurate prediction model for the specific capacity of organic anode active materials of lithium-ion batteries.^53^ On the other hand, some chemically significant descriptors were not extractable only from the weight diagram. In such a case, the descriptors were manually added for the model. For example, the yield prediction model was constructed by this scheme.^16^ However, it is not easy to quantify the physical significance, not just the correlation, of the variables based on chemical insights. In ES-BMA, such physical meaning is represented by the probability value (*p*). However, *p* is not estimated from the small data, as shown in Fig. 4d and e. The development of a new quantitative method is required to extract and select the more significant descriptors quantitatively.

### 2.5. Advantages of SpM-S compared with other algorithms

In linear regression models, ES-LiR contributes to providing accurate models using weight diagrams compared with other algorithms, such as LASSO with variable selection and multiple linear regression (MLR) without variable selection. In ES-LiR, linear regression models are exhaustively prepared using all the possible combinations of *x*_*n*_. The potential models are selected based on weight diagrams visualizing the contribution of each coefficient. As MLR-based models include the irrelevant *x*_*n*_, there may be an overfitting that causes a lowering of the prediction accuracy and generalizability. In the present study, the variable selection problem involved a relatively small number of variables. It is feasible to arduously search all the possible combinations and directly identify the optimal model.^47^ As other algorithms with variable selection construct specific models with certain CVE values based on approximations, the appropriate variables may not be extracted particularly in noisy and small training data. In our previous works,^45,52,53^ prediction models were constructed by LASSO to compare the prediction accuracy, which was calculated by five-fold CVE. The models using LASSO generally showed larger RMSE values to the test data compared with those using SpM-S, even though smaller RMSE values were achieved from some training data. The other modeling techniques use certain approximations to reduce the calculation cost. On the other hand, ES-LiR is applicable to small data at a realistic calculation cost. More accurate descriptors can be selected with our chemical insights from all the possible regression models.

The accuracy, generalizability, and interpretability of the sparse linear models based on small data were compared with those of other nonlinear algorithms in our previous works.^45^ The results imply that nonlinear models have concerns about overtraining linked to the training data and a lowering of the generalizability, particularly in the case of small data. In such a case, linear models are preferable to describe the whole trend of the data. We recognize the importance of frameworks, such as sure independent screening and sparsifying operator (SISSO),^62^ which integrates sure independent screening (SIS) with LASSO-based variable selection to efficiently manage ultra-high-dimensional descriptor spaces. However, in our current study, the dimensionality of the descriptor space was limited to several tens of dimensions, enabling an exhaustive search approach rather than requiring dimensionality reduction using SIS. Furthermore, as demonstrated in a recent work,^63^ a Monte Carlo-based approximate exhaustive search method could be employed for moderately high-dimensional scenarios. Our ongoing research efforts are directed toward integrating SIS and exhaustive search strategies to enhance the descriptor selection efficiency and effectiveness, particularly in high-dimensional and correlated descriptor spaces.

## 3. Conclusions

Linear regression prediction models for the yield, size, and size distribution of exfoliated nanosheets were constructed by SpM-S combining ES-LiR and domain knowledge on small data. The present work validated the model construction method and process, such as the significance of the data size, visualization method, and use of chemical insights for the variable selection. Weight diagrams were constructed that visualized the significance of the variables by color. Then, the significant descriptors were selected with the assistance of our chemical insight. The appearance of the weight diagram was changed with reducing the data size. The data size had a specific lower limit to extract the same appropriate descriptors. This method can be widely applied to validate whether the data size is sufficient or not. Whereas conventional ML algorithms with variable selection focus on a certain model with the minimum CVE value, the weight diagram of ES-LiR overviews a large number of models in ascending order of the CVE values. The visualization range of the CVE values had an effect on the appearance of the weight diagram leading to the extraction of the descriptors. A clear weight diagram suitable for the variable selection was obtained within about top 10% of the CVE rank, which was consistent with the one standard error rule. When the variables were selected using the probability of ES-BMA without the assistance of our domain knowledge, the descriptors could not be extracted only from the probability value. The fact implies that the domain knowledge could be effectively used for the variable selection to supplement the deficiency of data. The present study supports the validity of the prediction models for the yield, size, and size distribution of exfoliated nanosheets reported in our previous works. Moreover, SpM-S combining ES-LiR and chemical insight is a suitable method for small data in a variety of fields. In the present work, the models were constructed based on the data about the exfoliation of layered inorganic–organic composites into surface-modified nanosheets, as shown in Fig. 1a and b. The yield prediction model has also been applied to the exfoliation of other layered compounds, such as graphite and layered organic polymers.^64^ As the chemical features of the layer surface and dispersion media are used as the descriptors, the models can be applied to other types of the layered materials.

## Conflicts of interest

There are no conflicts to declare.

## Supplementary Material

NA-007-D5NA00215J-s001

NA-007-D5NA00215J-s002

## Data Availability

The data supporting this article have been included as part of the ESI.†
