# Supplementary material for: Data-scientific validation of prediction models for the controlled syntheses of exfoliated nanosheets
Source: Nanoscale Adv. 2025 Jun 4;7(15):4620–7. doi: 10.1039/d5na00215j (PMC12177519; doi:10.1039/d5na00215j)
Supplement: NA-007-D5NA00215J-s001 [file NA-007-D5NA00215J-s001.pdf]

Supplementary Information (SI) for Nanoscale Advances.
This journal is © The Royal Society of Chemistry 2025

Electronic Supplementary Information (ESI)

Data-scientific validation of prediction models for controlled syntheses of exfoliated nanosheets

Yuka Kitamura,[a] Yuki Namiuchi,[b] Hiroaki Imai,[a] Yasuhiko Igarashi*[b], Yuya Oaki*[a]

[a] Department of Applied Chemistry, Faculty of Science and Technology, Keio University, 3-14-1 Hiyoshi, Kohoku-ku, Yokohama 223-8522, Japan

[b] Institute of Engineering, Information and Systems, University of Tsukuba, 1-1-1 Tennodai, Tsukuba 305-8573, Japan

E-mail: oakiyuya@applc.keio.ac.jp, igayasu1219@cs.tsukuba.ac.jp

Contents

Methods P. S2

Datasets (Tables S1–S3) † P. S3

Weight diagrams with reducing the data size for y_1–y_3 (Figs. S1–S3) P. S5

†**Note**: All the datasets (Tables S1–S3) are available as csv file in a Zip archive.

Methods

Preparation of the reduced datasets: The total six different datasets were prepared from the original datasets with the random sorting ($i = 0, 1, 2, 3, 4, 5$). The number of y (N) was decreased step-by-step for each dataset. For example, the original 30 y_1 was reduced to 25 y_1 with the subtraction of five y_1. Then, the reduced datasets with 20 y_1 were prepared with the further subtraction of 5 y_1. The data was reduced in this manner. This reduction scheme was applied to total six datasets.

ES-LiR: In ES-LiR, the linear-regression models were constructed for all 2^j–1 combinations of x_n ($j = n$), such as {x_n only}, {x_1, x_2}, {x_1, x_3}, {x_1, x_4}, …, {x_2, x_3},….,{x_1, x_2, x_3}, {x_1, x_2, x_4}, … {x_1, x_2, …, x_n} with five-fold cross validation. In the present work, five-fold was used to reduce the time and calculation cost. The prediction accuracy of each model is evaluated using cross validation error. This algorithm was implemented in Python. The results are summarized in the weight diagram.

ES-BMA: In ES-BMA, the uncertainty for all 2^n combinations of variables is considered. A method of quantitatively evaluating the confidence level of feature selection was introduced using a weighted average of the model posterior probabilities, which is called BMA.[61] This method enables to evaluate the confidence level of feature selection and quantify the importance evaluation of features, which has been a qualitative one when using the weight diagram of the exhaustive search method. Furthermore, this approach quantitatively assesses the plausibility of descriptors as features. The summation over all combinations of indicator vectors can be calculated using the result of the exhaustive search, which is called ES-BMA.[61] The code used in this paper is available at https://github.com/okada-lab/exhbma.

Datasets

Training dataset for y_1 (yield)

In the folder of Tables S1-S3 Dataset, Table S1 (CSV file) is the original dataset for y_1.
The reduced datasets were included in the folder named as 'Table S1 _reduced-datasets_yield.' Each reduced dataset (CSV file) is named as 'yield_training_sample*N*-i (i = 0, 1, 2, 3, 4, 5)', where N is the number of y.

Training dataset for y_2 (lateral size)

In the folder of Tables S1-S3 Dataset, Table S2 (CSV file) is the original dataset for y_2.
The reduced datasets were included in the folder named as 'Table S2 _reduced-datasets_size.' Each reduced dataset (CSV file) is named as 'size_training_sample*N*-i (i = 0, 1, 2, 3, 4, 5)', where N is the number of y.

Training dataset for y_3 (size distribution)

In the folder of Tables S1-S3 Dataset, Table S3 (CSV file) is the original dataset for y_3.
The reduced datasets were included in the folder named as 'Table S3 _reduced-datasets_size-distribution.' Each reduced dataset (CSV file) is named as 'size-distribution _training_*N*-i (i = 0, 1, 2, 3, 4, 5)', where N is the number of y.

In Tables S1–S3, the following abbreviations were used to represent the host, guest, and dispersion medium. The detailed information was in our previous works.[28,34–36]

[Host]
TiO2: layered titanate
BST: layered tantalate
Co(OH)2: layered cobaltate
MnO2: layered manganate
Nb6O17: layered niobate

[Guest]
C_{14}-NH: tetradecylamine
NH_2-BA: 4-aminobenzylamine
CH3-BA or MeBA: 4-methylbenzylamine
FBA: 4-fluorobenzylamine
BA: benzylamine
OH-BA: 4-(aminomethyl)phenol

NEA: DL-1-(1-naphthyl)ethylamine
TpCNH2: 3-thiophenemethylamine
PEA: 2-(2-aminoethyl)pyridine
PhMe: *p*-tolylacetic acid
HQCA: 2,5-dihydroxybenzoic acid
C6-COOH: heptanoic acid
AQS: sodium anthraquinone-2-sulfonate monohydrate
C18-NH2: stearylamine
C6-NH2: hexylamine
API: 1-(3-aminopropyl)imidazole
DAMN: diaminomaleonitrile
BXPA or PhBx: 4-butoxyphenylacetic acid
OMe-BA: 4-methoxybenzylamine

[Dispersion medium]
NMP: *N*-methyl-2-pyrrolidone
DMSO: dimethyl sulfoxide
DMF: *N*,*N*-dimethylformamide
THF: tetrahydrofuran
DEG: diethylene glycol

Weight diagrams with reducing the data size for y_1–y_3

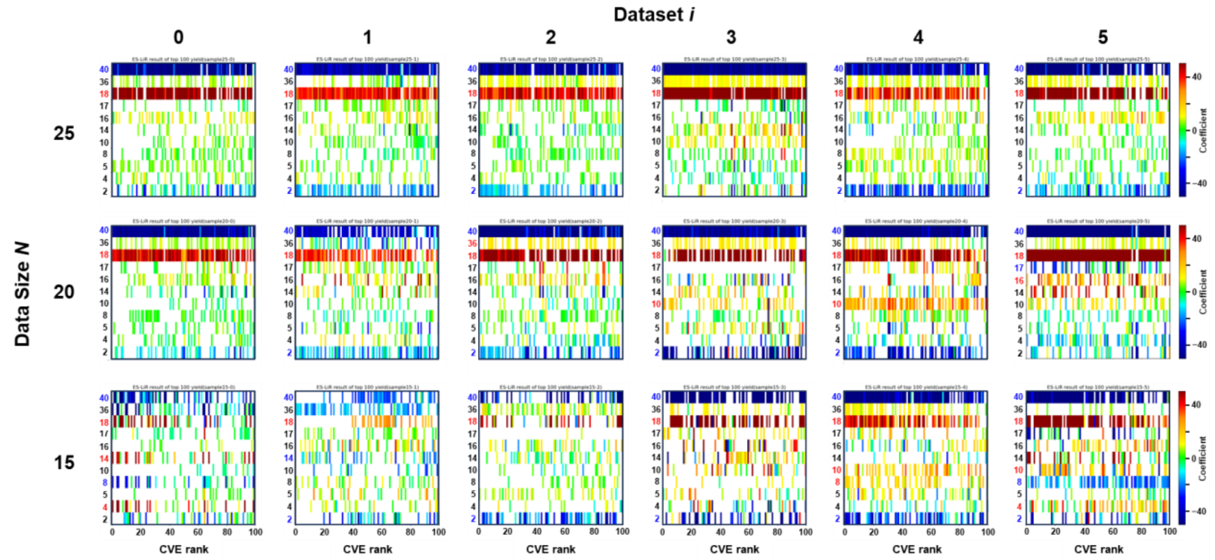

Fig. S1. Weight diagrams for y_1 with reducing the data size (N: the number of y_1). The number of the datasets (i = 0–5) corresponding to the file name, as mentioned in the Datasets section.

The descriptors were visually extracted from these weight diagrams. The number of the correct and incorrect x_n was counted to prepare Fig. 2f–h.

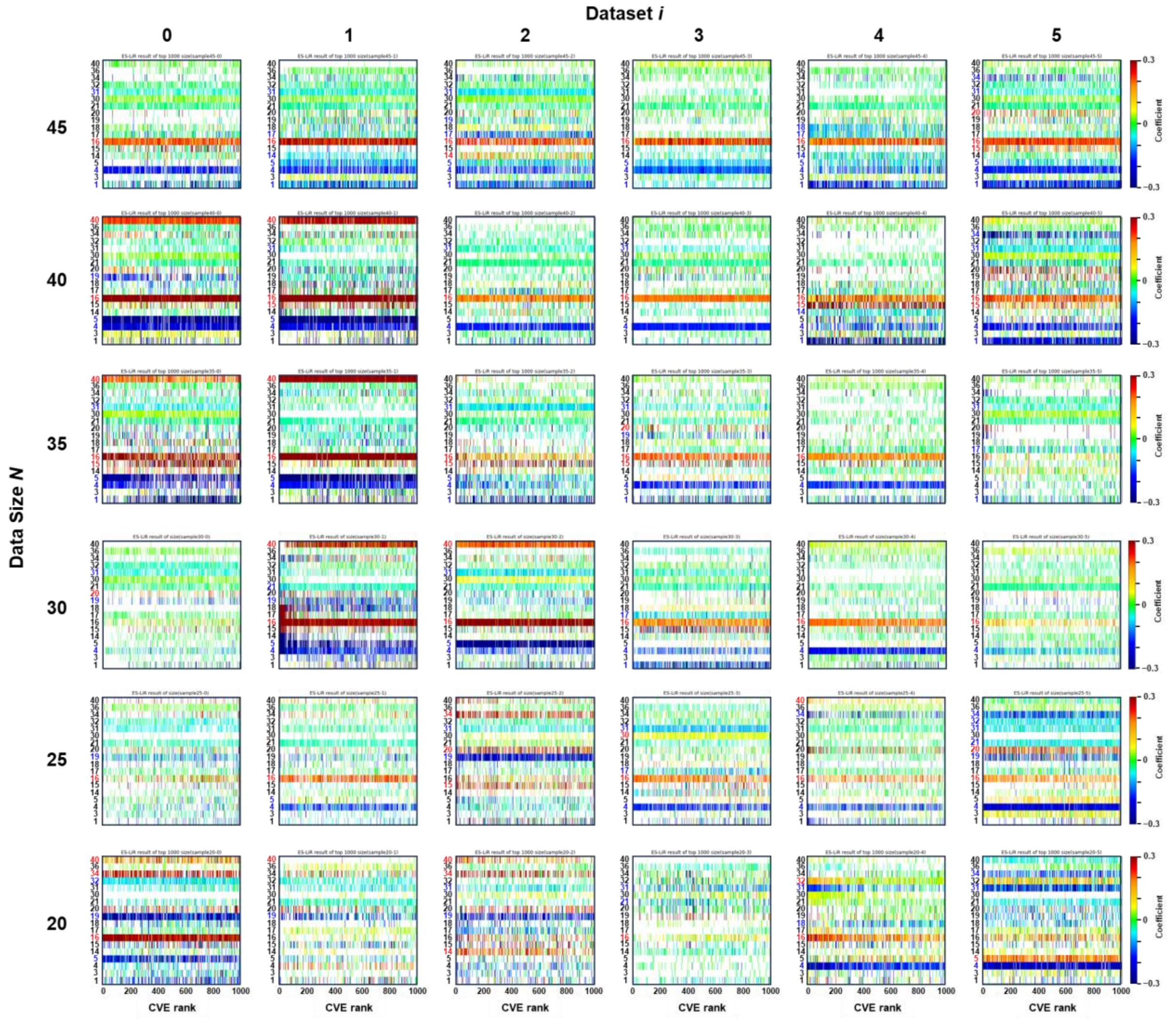

Fig. S2. Weight diagrams for y_2 with reducing the data size (N: the number of y_2). The number of the datasets (i = 0–5) corresponding to the file name, as mentioned in the Datasets section.

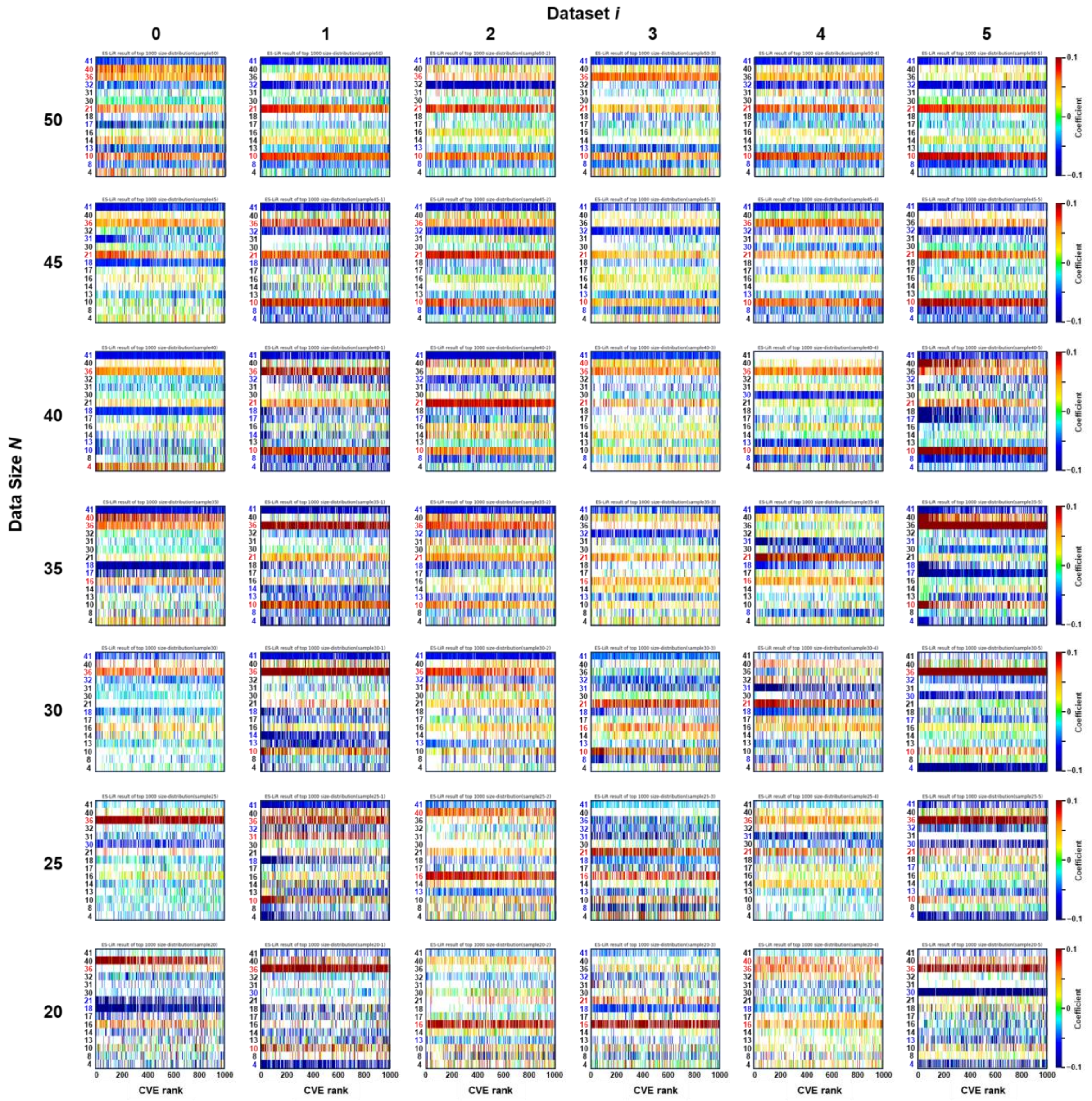

Fig. S3. Weight diagrams for y_3 with reducing the data size (N: the number of y_3). The number of the datasets (i = 0–5) corresponding to the file name, as mentioned in the Datasets section.
